# SET Domain Group 703 Regulates Planthopper Resistance by Suppressing the Expression of Defense-Related Genes

**DOI:** 10.3390/ijms241613003

**Published:** 2023-08-21

**Authors:** Peizheng Wen, Jun He, Qiong Zhang, Hongzhi Qi, Aoran Zhang, Daoming Liu, Quanguang Sun, Yongsheng Wang, Qi Li, Wenhui Wang, Zhanghao Chen, Yunlong Wang, Yuqiang Liu, Jianmin Wan

**Affiliations:** 1 State Key Laboratory of Crop Genetics & Germplasm Enhancement and Utilization, Jiangsu Nanjing Rice Germplasm Resources National Field Observation and Research Station, Jiangsu Provincial Research Center of Plant Gene Editing Engineering, Nanjing Agricultural University, Nanjing 210095, China; 2017201048@njau.edu.cn (P.W.); hj@njau.edu.cn (J.H.); 2021101104@stu.njau.edu.cn (Q.Z.); 2021101093@stu.njau.edu.cn (H.Q.); ran17854283133@163.com (A.Z.); liudm0924@163.com (D.L.); 2022201049@stu.njau.edu.cn (Q.S.); a1442216643@163.com (Y.W.); 2019201069@njau.edu.cn (Q.L.); 2018101088@njau.edu.cn (W.W.); c2812536935@outlook.com (Z.C.); yunlongwang@njau.edu.cn (Y.W.); 2 National Key Facility for Crop Gene Resources and Genetic Improvement, Institute of Crop Science, Chinese Academy of Agricultural Sciences, Beijing 100081, China

**Keywords:** rice (*Oryza sativa*), brown planthopper (BPH), histone methyltransferase, insect resistance

## Abstract

Plant defense responses against insect pests are intricately regulated by highly complex regulatory networks. Post-translational modifications (PTMs) of histones modulate the expression of genes involved in various biological processes. However, the role of PTMs in conferring insect resistance remains unclear. Through the screening of a T-DNA insertion activation-tagged mutant collection in rice, we identified the mutant *planthopper susceptible 1* (*phs1*), which exhibits heightened expression of SET domain group 703 (SDG703). This overexpression is associated with increased susceptibility to the small brown planthopper (SBPH), an economically significant insect pest affecting rice crops. *SDG703* is constitutively expressed in multiple tissues and shows substantial upregulation in response to SBPH feeding. SDG703 demonstrates the activity of histone H3K9 methyltransferase. Transcriptomic analysis revealed the downregulation of genes involved in effector-triggered immunity (ETI) and pattern-triggered immunity (PTI) in plants overexpressing *SDG703*. Among the downregulated genes, the overexpression of *SDG703* in plants resulted in a higher level of histone H3K9 methylation compared to control plants. Collectively, these findings indicate that SDG703 suppresses the expression of defense-related genes through the promotion of histone methylation, consequently leading to reduced resistance against SBPH. The defense-related genes regulated by histone methylation present valuable targets for developing effective pest management strategies in future studies. Furthermore, our study provides novel insight into the epigenetic regulation involved in plant-insect resistance.

## 1. Introduction

Plants have developed intricate defense mechanisms to protect themselves against insect pests [1]. Rice serves as a staple food for over half of the global population [2,3]. However, rice production faces significant threats from a range of insect pests. Among them, the most destructive insect pests for rice include the brown planthopper (*Nilaparvata lugens* Stål, BPH), the white-backed planthopper (*Sogatella furcifera* Horvath, WBPH), and the small brown planthopper (*Laodelphax striatellus* Fallén, SBPH) [4]. Although these three kinds of planthopper are all delphacid piercing-sucking pests, some studies found that BPH- and WBPH-susceptible varieties displayed resistance to SBPH, and few genes derived from a single resistance resource controlling broad spectrum resistance to the three rice planthoppers shared the same resistance locus [5,6]. These results indicate that the mechanisms underlying the resistance against the three kinds of rice planthoppers should be distinguished.

To date, nine resistance genes against BPH have been successfully cloned [7,8,9,10,11,12,13,14,15]. The majority of cloned BPH resistance genes are members of the leucine-rich repeat (LRR) family or surface-localized pattern-recognition receptors [16]. Notably, *Bph14*, *Bph9*, and their allelic variants encode intracellular nucleotide-binding leucine-rich repeat-containing receptors (NLRs), enabling them to activate effector-triggered immunity (ETI) by sensing effectors derived from insect feeding [7,11,17]. Additionally, the broad-spectrum and durable resistance gene, *Bph3*, comprises a gene cluster consisting of three genes that encode plasma membrane-localized lectin receptor kinases (OsLecRK1–OsLecRK3). These receptors serve as cell surface pattern recognition receptors (PRRs) to initiate pattern-triggered immunity (PTI), thereby mediating resistance against insects [9]. Thus, the above-mentioned studies demonstrate that rice possesses a dual-layered immune system (ETI or PTI) that facilitates effective defense against pests, resembling the disease resistance mechanisms observed in plants [16,18].

While several BPH resistance genes have been cloned, no resistance genes against SBPH or WBPH have been identified. SBPH not only inflicts direct damage on crops but also serves as a vector for devastating viral diseases, including rice stripe virus (RSV) and rice black-streaked dwarf virus (RBSDV) [19,20]. However, the understanding of the molecular mechanisms responsible for planthopper resistance is still limited.

Histone PTMs play a crucial role in chromatin modulation and serve as a fundamental mechanism for regulating gene transcription [21,22]. Most histone modifications have been identified on histone H3. Histone H3 methylation occurs at various lysine sites, including H3K4, H3K9, H3K27, and H3K36 [23]. These modifications typically exhibit a strong correlation with gene expression levels. Generally, trimethylation of H3K4 and K36 is associated with transcription initiation, whereas H3K9me2 and K27me3 are commonly linked to transcriptional suppression in plants [24].

SUVH proteins, homologous to SU(VAR)3–9, function as H3K9-specific methyltransferases and play a crucial role in chromatin function and development [24,25]. Numerous SUVH proteins have been identified in the genomes of plants [26]. For instance, *Arabidopsis* and rice contain 10 and 12 SUVH genes, respectively [27,28]. In *Arabidopsis*, *SUVH4*, *SUVH5*, and *SUVH6* are responsible for locus-specific mono- and di-methylation of H3K9 [29,30,31,32]. In rice, the SUVH protein SDG714 regulates the H3K9me2 level of a *copia*-like retrotransposon called *Tos17* and a series of genes that are vital for plant growth and development [33,34]. *SDG728* plays a positive regulatory role in the levels of H3K9me2 and H3K9me3, which are crucial for seed size and weight development [28]. However, the functions of most SUVH genes in rice remain unclear.

Previous studies have demonstrated that histone H3 methylation in plants is involved not only in regulating diverse developmental processes but also in pathogen defense responses [35,36]. For instance, in *Arabidopsis*, the basal level of H3K36me3, regulated by SDG8, contributes to the basal transcription of R genes that encode TIR-NB-LRR and CC-NBS-LRR proteins, leading to localized host cell death and peptide-triggered immunity [37,38]. Additionally, H3K4me3 regulates the locus-specific expression of TIR-NB-LRR genes such as *SNC1* and *RPP4* in *Arabidopsis*, thereby contributing to the plant’s basal defense [39]. These studies have revealed the significant role of histone H3 methylation in regulating plant immunity against pathogens. Notably, NLR genes also participate in plant defense responses against herbivorous insect infestations [16]. This observation suggests that histone H3 methylation could potentially be involved in plant resistance to insects. However, there have been no reports on histone methylation or SUVH proteins involved in plant resistance responses to insects.

In this study, we discovered that the histone H3K9 methyltransferase SDG703 plays a negative regulatory role in SBPH resistance in rice. T-DNA activation-tagged mutant line *phs1* and *SDG703* overexpressing lines exhibited greater susceptibility to planthoppers than the wild type, Dongjin. Subsequent investigations revealed that SDG703 downregulates the expression of ETI-related defense response genes and PTI-related cell surface receptor signaling pathway genes through histone methylation modifications, thereby suppressing planthopper resistance. These findings offer novel insight into the role of histone methylation modification in regulating plant resistance responses to insects, thereby expanding the understanding of host–insect interactions.

## 2. Results

### 2.1. Identification of Planthopper Susceptible Mutant phs1 

**Figure 1:**
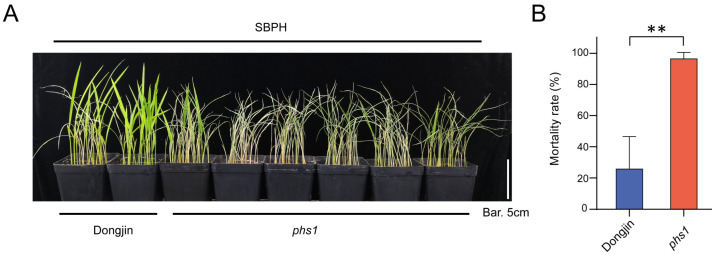
Mutant *phs1* displays more susceptibility to SBPH than the wild type, Dongjin. (**A**) Phenotypes of wild type (Dongjin) and mutant *phs1* post infestation with SBPH. Bar: 5 cm. (**B**) The seedling mortality rate of wild type and mutant *phs1* infested by SBPH. Significant differences were determined using the Student’s *t*-test. ** *p* < 0.01.

To investigate the molecular mechanism underlying the interaction between rice and SBPH, we employed a seedling box screening test to screen a collection of rice mutants with activation-tagged T-DNA insertion from the rice mutant library in South Korea [40]. Among them, one mutant (PFG_1A-14509), named *planthopper susceptible 1* (*phs1*), exhibited increased susceptibility to SBPH compared to the wild type, Dongjin (Figure 1A,B).

To validate the function of T-DNA insertion, T-DNA flanking sequences were determined by searching the Rice Functional Genomic Express database “http://signal.salk.edu/cgi-bin/RiceGE” (accessed on 20 September 2015). Flanking specific primers were designed for amplifying fragments and conducting sequencing analysis. Sequence alignment and PCR analysis confirmed the insertion of T-DNA at a position 144 bp upstream of the *Os04g0544100* translational initiation site (Figure S1A,B). Gene *Os04g0544100* encodes a histone lysine methyltransferase, SDG703, which is specific to H3K9. RT-qPCR analysis revealed that the expression of *SDG703* in mutant *phs1* was significantly increased by over 6-fold compared to that in Dongjin. However, the expression of *Os04g0544200*, *Os04g0544000*, and *Os04g0543900*, which are located upstream and downstream of *SDG703*, remained unchanged (Figure S2A,B). Collectively, these results demonstrated that T-DNA insertion led to an increase in the expression of *SDG703*. Furthermore, the upregulated transcription of *SDG703* in mutant *phs1* might potentially weaken the plant’s resistance against SBPH.

### 2.2. SDG703 Negatively Regulates SBPH Resistance in Rice

**Figure 2:**
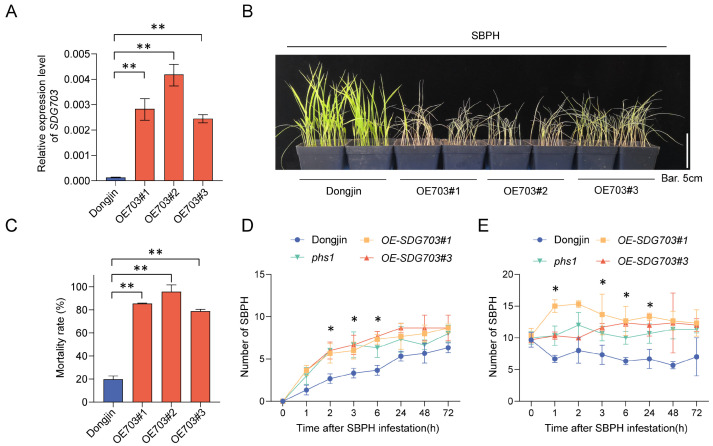
Overexpressing *SDG703* significantly reduces rice resistance to SBPH. (**A**) RT-qPCR analysis of *SDG703* in wild type (Dongjin) and three independent *SDG703* overexpressing transgenic lines, with *18s-rRNA* as the internal reference gene. (**B**) Phenotypes of wild type and three *SDG703* overexpressing transgenic lines (*OE703#1*, *OE703#2*, and *OE703#3*) subjected to SBPH infestation. Bar: 5 cm. (**C**) The seedling mortality rate of wild type and three independent *OE-SDG703* transgenic lines. (**D**) Dynamic changes in SBPH populations on wild type, mutant *phs1*, and *SDG703* overexpressing transgenic lines surrounded by SBPH sources. (**E**) Dynamic changes in SBPH populations on wild type, mutant *phs1*, and *OE-SDG703* transgenic lines initially infested with 10 SBPH. Data are shown as mean ± SEM (n ≥ 3). * *p* < 0.05, ** *p* < 0.01 using the Student’s *t*-test in (**B**–**D**).

To elucidate the role of *SDG703* in SBPH resistance, *SDG703* was overexpressed in the wild-type background, generating three independent transgenic lines designated as *OE-SDG703* (*OE703#1*, *OE703#2*, and *OE703#3*). RT-qPCR analysis revealed that the transcript levels of *SDG703* were increased over 18-fold in the three transgenic lines compared to that in the wild type (Figure 2A). Subsequently, the SBPH resistance of the three overexpressing lines was evaluated using a modified seedling box screening test. All three *OE-SDG703* lines exhibited increased susceptibility to SBPH compared to the wild type, consistent with mutant *phs1* (Figure 2B,C). Additionally, we generated two *SDG703* knockout mutants using the CRISPR/Cas9 strategy (Figure S3A). Two knockout lines, namely *KD-SDG703#1* with a two-base pair insertion at the target site and *KD-SDG703#2* with a two-base pair deletion at the target site, were obtained. Both base pair insertion and deletion resulted in premature termination of transcription. However, the SBPH resistance levels of the two *SDG703* knockout lines were not significantly different from that of the wild type (Figure S3B,C).

To assess the antixenotic effect of mutant *phs1*, *OE-SDG703*, and the wild type on SBPH nymphs, we conducted the experiment in two ways. In the initial assay, the results revealed a steady increase in SBPH populations on *OE-SDG703*, mutant *phs1*, and the wild type over time. The numbers of SBPHs on mutant *phs1* and *OE-SDG703* were significantly higher than those on the wild type at 2, 3, and 6 h after exposure to SBPH (Figure 2D). In the second assay, the results indicated a notable increase in SBPH populations on mutant *phs1* and *OE-SDG703* compared to those on the wild type after 1, 3, 6, and 24 h of SBPH infestation (Figure 2E). These results suggested that SBPH exhibited a preference for feeding on mutant *phs1* and *OE-SDG703* plants over the wild type, potentially explaining their reduced resistance to SBPH. Collectively, all of these results indicated the negative regulatory role of *SDG703* in SBPH resistance.

### 2.3. SDG703 Is Widely Expressed in Various Plant Tissues

**Figure 3:**
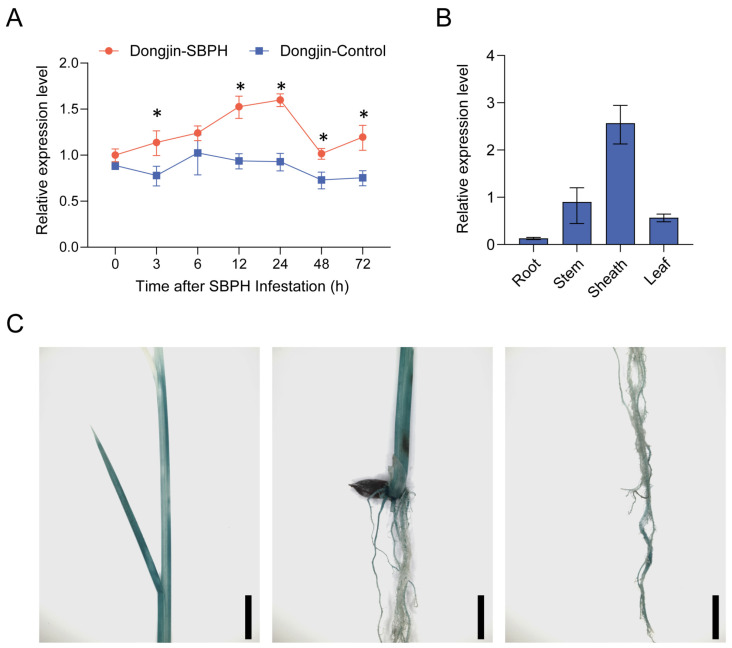
Expression pattern analysis of *SDG703*. (**A**) Transcriptional analysis of *SDG703* in response to SBPH infestation. *18s-rRNA* was used as the internal reference gene. The asterisks indicate significant differences using the Student’s *t*-test. * *p* < 0.05 (n ≥ 3). (**B**) Tissue expression of *SDG703* in seedling roots, stem, sheath, and leaves. *18s-rRNA* was used as the internal reference gene. (**C**) Expression pattern of the reporter *β-GLUCURONIDASE* (*GUS*) gene driven by the SDG703 promoter (*SDG703pro::GUS*) in rice seedlings. GUS activity was detected in the stem, sheath, roots, and leaves. Bar: 1 cm.

To elucidate the biological role of *SDG703*, we initially investigated its expression patterns in rice. RT-qPCR analysis revealed a significant induction of *SDG703* transcript levels in response to SBPH feeding (Figure 3A). Furthermore, *SDG703* was constitutively expressed in all examined organs of rice plants, including the roots, stem, sheath, and leaves of seedlings. Remarkably, *SDG703* exhibited high expression levels in the sheath, which is typically the site of SBPH feeding (Figure 3B).

To further investigate the expression pattern of *SDG703*, we generated transgenic rice plants (*SDG703pro::GUS*) that expressed GUS under control of the *SDG703* promoter. Histochemical staining revealed GUS activity in all of the examined organs, including the leaves, stem, sheath, and roots (Figure 3C). This observation was confirmed by RT-qPCR analysis, which detected a higher abundance of *SDG703* transcripts in the sheath of rice seedlings.

**Figure 4:**
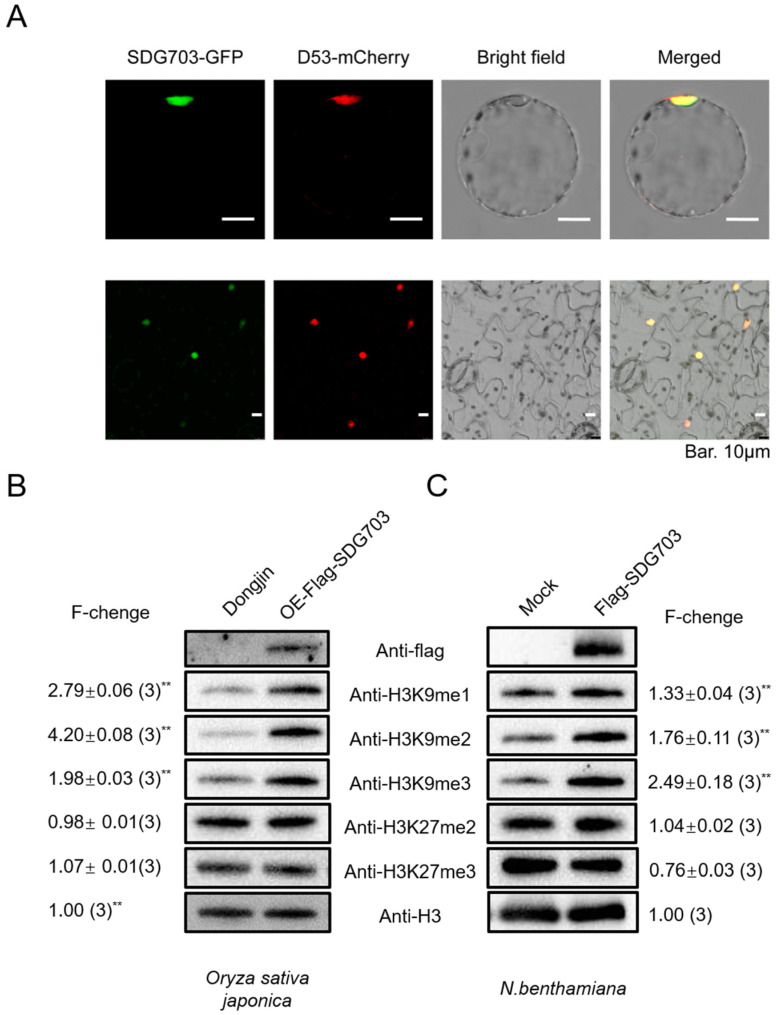
Expression pattern analysis of *SDG703*. (**A**) Subcellular localization of SDG703 in rice protoplasts and *Nicotiana benthamiana* leaf epidermal cells. D53-mCherry was used as the nuclear marker. Bar: 10 µm. (**B**) Western blot analysis of H3K9 and H3K27 methylation in *OE-Flag-SDG703*. The fold change (F-change) measures the ratio of signal intensity of *OE-Flag-SDG703* with the wild type (set as 1) and is shown as mean ± SE from three replicates, after normalization using H3. The asterisks indicate statistically significant F-changes using the Student’s *t*-test, ** p < 0.01. (**C**) Western blot analysis of H3K9 and H3K27 methylation in *Nicotiana benthamiana* transient expression. The fold change (F-change) measures the ratio of signal intensity of *Flag-SDG703* with Mock (set as 1) and is shown as mean ± SE from three replicates, after normalization using H3. The asterisks indicate statistically significant F-changes using the Student’s *t*-test, ** p < 0.01.

To ascertain the subcellular localization of SDG703, we transiently co-expressed *SDG703-GFP* and a nucleus localization marker, *D53-mCherry*, in rice protoplasts and *Nicotiana benthamiana* leaves, respectively. In both rice protoplasts and *Nicotiana benthamiana* leaves, the SDG703-GFP fusion protein exhibited co-localization with the nuclear marker, D53-mCherry, supporting the predicted nuclear localization of SDG703 (Figure 4A).

### 2.4. SDG703 Contributes to Histone H3K9 Modification

To investigate the involvement of SDG703 in histone methylation, we generated plants overexpressing *flag-SDG703* (*OE-flag-SDG703*). The expression level of *SDG703* was increased by more than 20-fold compared to the wild type (Figure S4A). *OE-flag-SDG703* plants were also found to be more susceptible to SBPH compared to the wild type (Figure S4B,C). In the Western blot experiment, specific antibodies were used to detect the levels of histone methylation in *OE-flag-SDG703* and wild-type plants. The results revealed significant increases in H3K9me1, H3K9me2, and H3K9me3 levels in *OE-flag-SDG703*, while the levels of H3K27me2 and H3K27me3 were unaffected compared to those in the wild type.

To further confirm the function of SDG703 in H3K9 methylation, we also transiently expressed *flag-SDG703* in *Nicotiana benthamiana* leaves. Levels of H3K9me1, H3K9me2, and H3K9me3, but not H3K27me2 and H3K27me3, were significantly higher in *Nicotiana benthamiana* transiently expressing *flag-SDG703* than in control plants (Figure 4B,C). Collectively, these results indicated that SDG703 is involved in H3K9 methylation, and this function is conserved.

### 2.5. SDG703 Is Required for Proper Expression of a Set of Defense Response Genes

We investigated whether the difference in antixenosis to SBPH nymphs between *OE-SDG703* and the wild type was due to reinforcement of the sclerenchyma, a supportive structural tissue located adjacent to the vascular bundle, which hinders the insertion of stylets into phloem cells for feeding [41]. We evaluated lignin and cellulose accumulation in fresh leaf sheaths by staining them with phloroglucinol and fluorescent brightener. The staining intensity in *OE-SDG703* showed no significant difference from that in the wild type (Figure S5A,B). These results indicated that SDG703 has no effect on the biosynthesis of lignin and cellulose. Thus, the difference in antixenosis may be caused by other molecular mechanisms.

To further investigate the global impact of SDG703 involvement in the regulation of SBPH resistance, we conducted high-throughput transcriptome sequencing (RNA-seq) analysis of wild-type and *OE-SDG703* plants under normal conditions and SBPH infestation. For *OE-SDG703* plants, a total of 220 and 268 genes were differentially downregulated more than 2-fold compared to the wild type under normal conditions and SBPH infestation, respectively.

It is worth considering that the methylation of H3K9 is generally associated with gene transcriptional repression [42]. We hypothesized that the downregulation of 220 and 268 genes was likely a result of SDG703-mediated gene repression. Gene Ontology (GO) enrichment analysis indicated that the 220 downregulated genes under normal conditions were significantly associated with various biological processes, particularly the defense response and the cell surface receptor signaling pathway. Furthermore, the 268 downregulated genes under SBPH infestation were also significantly enriched in the defense response and the cell surface receptor signaling pathway (Figure S6).

**Figure 5:**
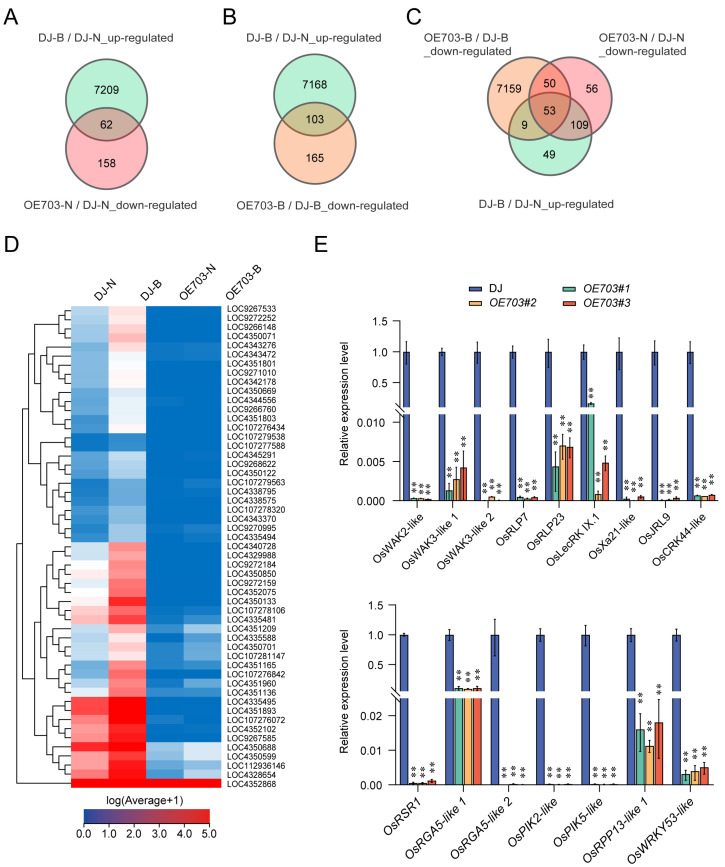
*SDG703* is required for proper expression of PRR and NLR genes in rice. (**A**) Venn diagram showing overlap of the upregulated genes in DJ-B vs. DJ-N and the downregulated genes in *OE703*-N vs. DJ-N. DJ-N: Dongjin without SBPH infestation. DJ-B: Dongjin with SBPH infestation. *OE703*-N: *OE-SDG703* without SBPH infestation. *OE703*-B: *OE703* with SBPH infestation. (**B**) Venn diagram showing overlap of the upregulated genes in DJ-B vs. DJ-N and the downregulated genes in *OE703*-B vs. DJ-B. (**C**) Venn diagram showing overlap of the upregulated genes in DJ-B vs. DJ-N, the downregulated genes in *OE703*-N vs. DJ-N, and the downregulated genes in *OE703*-B vs. DJ-B. (**D**) Heatmaps showing the genes upregulated by SBPH feeding in the wild type but downregulated in *OE-SDG703*. Each horizontal bar represents a single gene. Blue indicates a relatively low level of transcription, while red indicates a relatively high level of transcription. (**E**) Expression analysis of PRR and NLR genes in wild type and *OE-SDG703* under normal conditions by RT-qPCR. Values are the mean ± SD of three individual biological replicates, normalized to the internal control *18s-rRNA*. Significant differences were determined using the Student’s *t*-test. ** *p* < 0.01.

Venn diagram analysis revealed that 7271 genes were upregulated in the wild type upon SBPH infestation, suggesting their induction by SBPH. Under normal conditions and SBPH infestation, 62 and 103 differentially expressed downregulated genes in *OE-SDG703* plants, respectively, were found to overlap with the SBPH-induced genes in the wild type (Figure 5A,B). Furthermore, to identify genes that were potentially repressed by SDG703 at the structural level, Venn diagram analysis was performed on the 62 and 103 differentially expressed downregulated genes, resulting in an overlap of 53 genes (Figure 5C). It was hypothesized that these 53 differentially expressed downregulated genes were potentially direct targets of SDG703-mediated downregulation. The heatmap of the 53 differentially expressed genes confirmed that while most of these genes were significantly upregulated by SBPH infestation in the wild type, they were downregulated in *OE-SDG703* under both normal conditions and SBPH infestation (Figure 5D). Furthermore, a subset of these 53 genes, which were closely associated with plant defense, was selected from the *OE-SDG703* lines under SBPH infestation for further validation. The genes are listed in Table S2. The RT-qPCR analysis results of *OE-SDG703* and mutant *phs1* were consistent with the RNA-seq data (Figure 5E and Figure S7). The genes are listed in Table S3. These results suggested that SDG703 participates in the transcriptional downregulation of the tested defense-relative genes.

**Figure 6:**
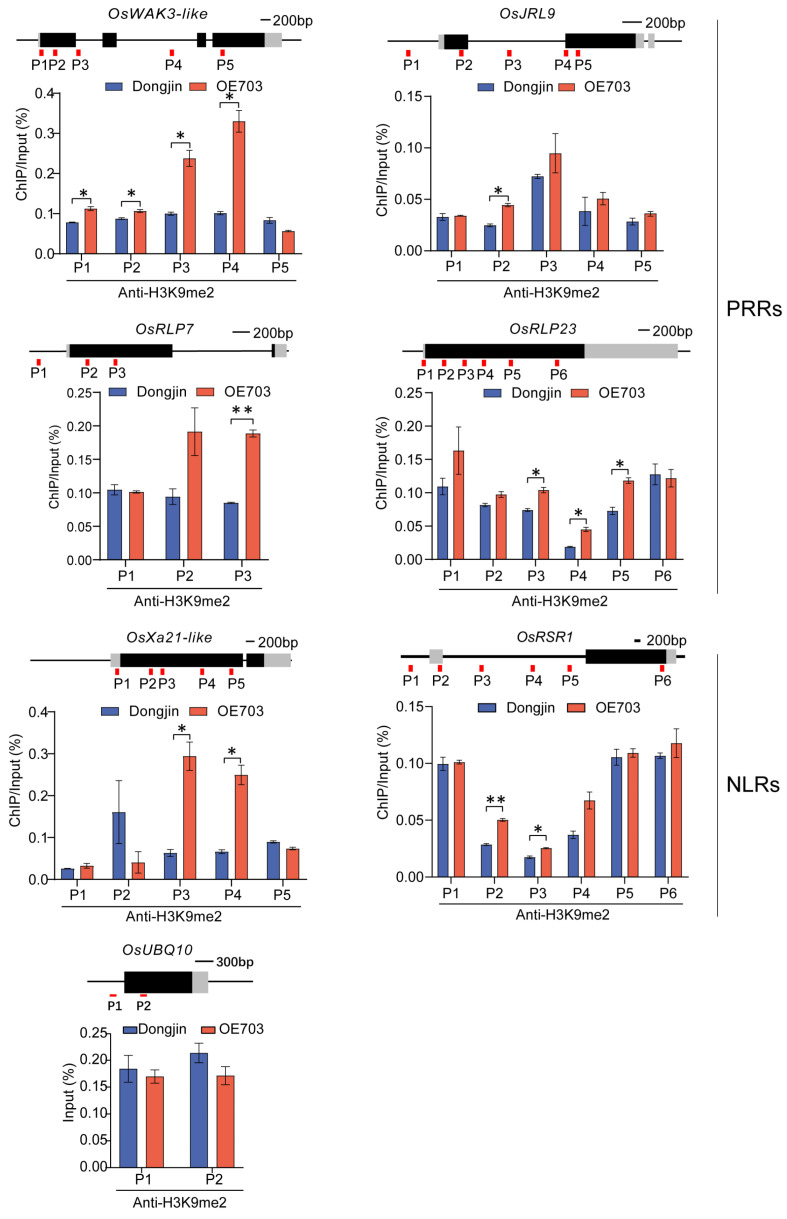
ChIP-qPCR for H3K9me2 modification of *NLRs* and *PRRs* downregulated by *SDG703*. The levels of H3K9me2 on *UBQ10* were used as the negative control. Data represent mean ± SD (n = 3). The asterisks represent statistically significant differences using the Student’s *t*-test, * p < 0.05, ** p < 0.01.

**Figure 7:**
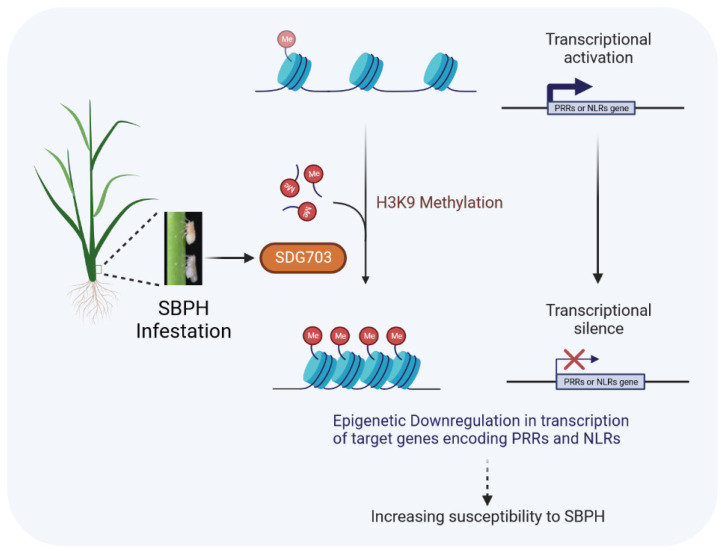
Work mode of SDG703 suppressing SBPH resistance in rice. SBPH infestation upregulates the transcriptional level of *SDG703*. SDG703 represses the transcriptional level of *NLRs* and *PRRs* by increasing H3K9me2 levels, resulting in the reduction of rice resistance against SBPH. Created with BioRender.com.

To investigate the association between SDG703-mediated H3K9 methylation and the downregulated defense response genes, chromatin immunoprecipitation (ChIP-qPCR) was performed using the commercially available anti-H3K9me2 antibody in *OE-SDG703* and the wild type. H3K9me2 has been detected across the gene body, and RT-qPCR primers were designed to target the gene body regions of these defense response genes, allowing for overlapping amplification. The results showed a significant increase in H3K9me2 levels of these defense response genes in *OE-SDG703* compared to the wild type (Figure 6). Therefore, these findings suggested that SDG703 likely represses the expression of resistance genes associated with the recognition of effector-triggered immunity (ETI) and pattern-triggered immunity (PTI) pathways through H3K9 methylation modification, thereby inhibiting resistance against SBPH in rice (Figure 7).

## 3. Discussion

Histone methylation is a fundamental mechanism for regulating gene transcription and plays a crucial role in a wide range of biological processes, including growth, development, and defense responses to various abiotic and biotic stresses in plants [43,44]. While numerous studies have explored the involvement of histone methylation in plant immunity to plant pathogens [36], the role of histone methylation in insect resistance remains unexplored. In this study, we discovered that the rice histone H3K9 methyltransferase gene *SDG703* negatively regulates SBPH resistance in rice and we provide evidence that this process is associated with the histone H3K9 methylation of defense-related genes. Thus, our findings expand the understanding of histone methylation modifications in the context of plant–insect interactions.

Plants have developed a two-layered immune system to defend against pathogens, initiated by pattern recognition receptors (PRRs) and nucleotide-binding leucine-rich repeat receptors (NLRs), leading to pattern-triggered immunity (PTI) and effector-triggered immunity (ETI), respectively [45]. Previous studies have shown that the majority of cloned brown planthopper (BPH) resistance genes belong to the PRR and NLR families [7,9,11,17], indicating the involvement of PTI and ETI in insect resistance in rice. In the present study, we observed the differential downregulation of numerous PRR and NLR genes in *OE-SDG703* under both non-infestation and infestation conditions compared to that in the wild type (Figure 5). These genes included ETI-associated genes such as *OsRSR1*, *RPP13-like 1*, *PIK-2-like*, and *RGA5-like 1*, as well as PTI-associated genes such as *WAK3-like 1*, *OsXa21-like*, *OsRLP7*, *OsRLP23*, *OsJRL9*, and *OsLecRK IX.1*. In a previous report, it was demonstrated that the disease resistance protein-encoding gene, *OsRPM1*, known as *OsRSR1*, is involved in sheath blight resistance, and the Jacalin-related lectin gene, *OsJRL9*, exhibited responsiveness to *Magnaporthe oryzae* challenge [46,47]. These findings indicated that *SDG703* may suppress the ETI and PTI pathways, thereby attenuating rice resistance against SBPH, suggesting similarity with the plant defense response to pathogens and insects. However, further studies are required to verify if ETI and PTI are also involved in SBPH resistance, similar to their role in rice resistance against BPH.

Previous studies have reported that most rice SUVH genes overexpressing or RNAi transgenic plants do not exhibit any noticeable phenotypic changes, except for *SDG728* RNAi plants, which show reduced seed size and weight [28]. Similarly, *SUVH4*, *SUVH5*, and *SUVH6* mutant in *Arabidopsis* do not exhibit any phenotypic changes [30,48]. Consistent with these findings, while overexpressing *SDG703* led to a significant reduction in rice SBPH resistance, knocking out *SDG703* did not affect SBPH resistance nor result in any significant differences in morphological phenotype. Collectively, these observations suggested that SUVH genes may exhibit functional redundancy in both plant development and the regulation of resistance response to insects.

In previous studies, numerous SUVH proteins have been identified as having methyltransferase activity targeting histone H3K9. For instance, *Arabidopsis* SUVH4, SUVH5, and SUVH6 have been shown to exhibit mono- or di-methyltransferase activity towards histone H3K9 [28,29]. Loss of function of *SUVH4* leads to a decrease in heterochromatic H3K9me2 levels, while the triple mutant (*suvh4*, *suvh5*, and *suvh6*) exhibits the loss of both H3K9me1 and H3K9me2 at target loc. In rice, the SUVH protein SDG728 positively regulates H3K9me2 and H3K9me3, while SDG713 specifically affects the H3K9me2 level [26]. Our findings revealed that SDG703 is involved in the modulation of mono-, di-, and tri-methylation of H3K9. These results provide evidence for the diverse functions of various SUVH proteins in H3K9 methylation.

DNA methylation is highly correlated with the histone H3K9me marker in silenced genes [49]. SUVH39-mediated H3K9 methylation directs DNA methylation to major satellite repeats at pericentric heterochromatin [50]. SUVH2 and SUVH9 are required for Pol V occupancy at RNA-directed DNA methylation loci [51]. In this study, we found that SDG703 promotes the histone methylation of defense-related genes and suppresses their expression. Further studies are needed to investigate that whether the transcript suppression of these defense-related genes caused by SDG703 is associated with DNA methylation.

## 4. Materials and Methods

### 4.1. Plant Materials and Growth Conditions

The T-DNA insertion activation-tagged mutant, *phs1* (PFG1A-14509), was obtained from the PFG T-DNA mutant database “http://signal.salk.edu/cgi-bin/RiceGE” in the *Oryza sativa* ssp. *Japonica* cv Dongjin background [40], originating from the Department of Plant Systems Biotechnology at Kyung Hee University, Korea. The insertion of T-DNA was identified through PCR using the specific primers listed in Table S1. *SDG703* overexpressing and knockout transgenic plants were also in the Dongjin background. Rice plants were cultivated in paddy fields under controlled conditions at 26 ± 2 °C with 50% relative humidity and a 16:8 light-to-dark photoperiod in an artificial climate room at Nanjing Agricultural University.

### 4.2. Planthopper Maintenance and Planthopper Resistance Evaluation

A colony of the small brown planthopper (SBPH) was collected from rice fields in Tuqiao Town, Nanjing City, Jiangsu Province (31°55′51″ N, 119°11′41″ E). The colony was maintained on the susceptible cultivar *Oryza sativa* L. *japonica* Suyunuo (SYN) under controlled conditions in an artificial climate room at Nanjing Agricultural University. The temperature was maintained at 26 ± 2 °C, with a relative humidity of 50% and a 16:8 light-to-dark photoperiod. The evaluation of SBPH resistance was conducted in a greenhouse. To ensure effective and accurate scoring of all test materials, the seedlings of the susceptible control were allowed to die based on the bulk seedling test [52]. The resistance to SBPH of all test materials was assessed using a slightly modified seedling box screening test described by Xu [53]. In this test, approximately 30 seeds from each individual plant were planted in a soil-filled plastic pot measuring 5.8 cm × 6.0 cm, with a hole at the bottom. At the 1.5- to 2.0-leaf stage, the seedlings were infested with second to third instar SBPH nymphs at a rate of 25 insects per seedling. Each test was replicated three times.

### 4.3. Antixenosis to Nymphs of SBPH in Rice Seedling

The experiment was conducted following previously described protocols with some modifications [9,54]. Briefly, 15 germinated seeds of the wild type (Dongjin), mutant *phs1*, and *OE-SDG703* lines were planted in a row with one seed per pot, maintaining a one-centimeter distance between the seeds. The plants were exposed to second to third instar SBPH nymphs at the 1.5- to 2.0-leaf stage. In the initial trial, the pots containing each line were infested with a heavily infested SYN plant as the source of SBPHs and placed in cages covered with nylon netting. The number of SBPHs on each variety was recorded at 1, 2, 3, 6, 24, 48, and 72 h after infestation. In the subsequent trial, the plants were arranged as described above, except that each plant was infested with 10 SBPH nymphs. The number of SBPHs on each variety was recorded at 1, 2, 3, 6, 24, 48, and 72 h after infestation. The experiments were repeated three times.

### 4.4. Vector Construction

The overexpression constructs were generated by amplifying the entire coding sequence regions of *SDG703* using gene-specific primers from the wild-type (Dongjin) seedling cDNA. The amplified PCR products were then inserted into *Kpn* Ⅰ- and *Bam*H Ⅰ-digested binary vectors, namely *pCUBI1309* and *pCAMBIA1300-221-Flag*, respectively, using the In-Fusion Advantage PCR Cloning Kit (Clontech, Mountain View, CA, USA). The integrity of all constructs was confirmed through DNA sequencing.

For the generation of the *SDG703* knockout lines, a 20-bp gene-specific sequence of *SDG703* was synthesized and annealed to form the oligo adaptors. Subsequently, the adaptors were cloned into the CRISPR-Cas9 expression vector *pOs-Cas9* [55,56].

To construct the *SDG703pro::GUS* plasmid, a 2000-bp fragment upstream of the *SDG703* start codon, corresponding to the *SDG703* promoter, was amplified using the primer pair SDG703pro-F/-R. The amplified PCR products were then inserted into *Hind* Ⅲ- and *Bam*H Ⅰ-digested binary vector *pCAMBIA1381Z*.

To transform *Nicotiana benthamiana*, the vector *pCAMBIA1305-GFP-SDG703* was constructed by PCR, amplifying the *SDG703* coding sequence from Dongjin seedling cDNA. The amplified product was then cloned downstream of the cauliflower mosaic virus (CaMV) 35S promoter in *Xba* Ⅰ- and *Bam*H Ⅰ-digested binary vector *pCAMBIA1305.1-GFP*.

To investigate the cellular localization of SDG703, the *SDG703* coding sequence was PCR-amplified from Dongjin seedling cDNA using the primers *pAN-SDG703*. The amplified product was inserted between the CaMV 35S promoter and the NOS cellular terminator in *Xba* Ⅰ- and *Bam*H Ⅰ-digested binary vector *pAN580*.

### 4.5. Plant Transformation

The recombinant plasmids were introduced into rice Dongjin calli using the *Agrobacterium*-mediated method. The hygromycin-resistant calli were cultured in an artificial incubator to generate transgenic plants. The T_0_ plants were cultivated in the paddy fields of Nanjing Agricultural University, and positive plants were confirmed using PCR and sequencing. T_1_ seeds were obtained through self-pollination, and positive T_2_ seedlings were used for evaluating SBPH resistance.

### 4.6. Cellular Observation of Sclerenchyma

Fresh hand-cut specimens were obtained from rice leaf sheaths at the tillering stage for cellular observation of lignin. These specimens were then fixed, sectioned, and stained using the phloroglucinol-HCl method (3% (wt/vol) phloroglucinol in ethanol:12 N HCl in a 1:2 ratio), as previously described [57]. The stained sections were examined under an Olympus BX51 microscope (Olympus Optical, Tokyo, Japan). A minimum of 20 sections were observed for each cultivar or line.

To observe cellulose at the cellular level, rice leaf sheaths at the tillering stage were carefully cut from seedlings using a razor blade. The cut samples were briefly rinsed in 0.1 M sodium phosphate buffer (pH 6.8) and then fixed in 2.5% glutaraldehyde in 0.1 M sodium phosphate buffer (pH 6.8). Subsequently, they were placed under vacuum overnight at low temperature (4 °C). After fixation, the samples were rinsed three times for 10 min each in 0.1 mol/L sodium phosphate buffer. The samples were then dehydrated in a series of ethanol concentrations (10%, 20%, 40%, 80%, and three changes of 100%) for 15 min at each concentration under low-temperature conditions (−20 °C). The dehydrated samples were embedded in resin (LR White; The London Resin Company, Basingstoke, UK) by gradually increasing the resin concentration to prevent tissue damage. Thin sections (1 μm) were obtained using a microtome (Leica RM2265, Wetzlar, Germany), transferred to a glass slide with a drop of water, and heated on a hot plate at 60 °C until the water evaporated. The sections were then stained with Fluorescent Brightener 28 (910090, Sigma-Aldrich, Darmstadt, Germany) and examined using a microscope (Leica TCS SP5) with the UV channel [58,59].

### 4.7. Tissue-Specific Expression Test Using GUS Staining

β-Glucuronidase activity was assessed using the Beijing Huayueyang GUSBlue KIT (GT0391, Beijing, China), following the established protocol [60]. Transgenic rice seedlings carrying the *SDG703pro::GUS* construct, which were seven days old and cultivated in a growth chamber under long-day (LD) conditions, were utilized for the analysis. Samples of leaves, stem, sheath, and roots were collected for staining.

### 4.8. Transient Expression in Nicotiana benthamiana

The *Agrobacterium* strain EHA105 carrying the recombinant vector was cultured overnight at 28 °C. The cells were subsequently resuspended and incubated in induction buffer (10 mM MES pH 5.6, 10 mM MgCl_2_, and 150 μM acetosyringone) at room temperature for 2 h prior to infiltration. For the co-infiltration experiments, the suspensions were mixed to achieve the desired final OD_600_ values and co-transformed with the virus-silencing suppressor, *Tombusvirus P19*, into the leaves of 5-week-old *Nicotiana benthamiana* plants using a needleless syringe. Transformation of *Nicotiana benthamiana* leaves with *Tombusvirus P19* was designated as Mock. Two days following infiltration, the plant leaves were utilized for the extraction of histone proteins.

### 4.9. Subcellular Localization Analysis

The expression constructs were co-transfected into rice protoplasts following previously described protocols [61] with some modifications. Briefly, 10 μg of plasmid DNA was mixed with 200 μg of protoplasts from each sample (approximately 1 × 10^6^ cells). A freshly prepared polyethylene glycol solution (220 μL; 40% (*w*/*v*) polyethylene glycol 4000, 0.2 M mannitol, and 0.1 M CaCl_2_) was added, and the mixture was incubated in the dark at room temperature for 20 min. After incubation, 1 mL of W5 solution was slowly added to the sample. The resulting solution was gently mixed by inverting the tube, and the protoplasts were pelleted by centrifugation at 1500 rpm for 3 min. The protoplasts were then gently resuspended in 1 mL of W5 solution. Finally, the protoplasts were transferred to multi-well plates and cultured at room temperature for 16 h under either light or darkness. The nuclear marker, mCherry-D53, a well-established fluorescent protein marker, was used [62]. Protoplasts were observed using a confocal laser scanning microscope (Leica TCS SP5).

### 4.10. RNA Extraction and RT-qPCR Analysis

Total RNA from the rice samples was extracted using the RNAprep Pure Plant Kit (TIANGEN Biotech, Beijing, China). First-strand cDNA synthesis was performed using the PrimeScript^®^ RT Kit (TaKaRa, Otsu, Japan). Real-time quantitative PCR (RT-qPCR) reactions were conducted on the BIO-RAD CFX96™ Real-Time System using the SYBR Premix Ex Taq RT-PCR Kit (TaKaRa). The relative expression level of each gene was determined using the 2^−ΔΔCt^ method, as previously described [63], with *18s-rRNA* (AH001749.2) [64] serving as the internal control. Each experiment was replicated three times. The primers used for RT-qPCR assays are listed in Table S1.

### 4.11. Plant Histone Protein Extraction and Western Blot Analysis

Two-week-old rice seedlings, cultured in a growth chamber under LD conditions, were ground to powder, and histones were extracted following the manufacturer’s protocol (BB31171, BestBio, Shanghai, China). The supernatant was boiled in 5 × SDS sample buffer (250 mmol/L Tris-HCl, pH 6.8, 10% SDS, 0.5% bromophenol blue, 50% glycerol, 5% β-mercaptoethanol). Protein extracts from plants were prepared as described above. Protein samples were separated on 10% SDS-PAGE gel and subsequently transferred to polyvinylidene fluoride fluoropolymer (PVDF) membranes (Millipore, Darmstadt, Germany). The membranes were blocked with 5% skimmed milk powder in PBST buffer (20 mmol/L Tris-HCl, pH 7.4, 150 mmol/L NaCl, and 0.05% [*w*/*v*] Tween 20) for 1 h at room temperature. The target protein bands were sequentially detected using antibodies against H3K9me (Ab9045, Abcam, Cambridge, UK), H3K9me2 (Ab1220, Abcam), H3K9me3 (Ab8898, Abcam), and H3 (Ab1791, Abcam), diluted at a ratio of 1:5000 with the dilution buffer (20 mmol/L Tris-HCl, pH 7.4, 150 mmol/L NaCl, 0.05% [*w*/*v*] Tween 20, and 5% [*w*/*v*] skimmed milk). Anti-H3 (Ab1791, Abcam) antibody was used as an internal reference. Anti-IgG (H+L chain) (Rabbit) pAb-HRP (H+L) (458, MBL, Beijing, China) and Anti-IgG (H+L chain) (Mouse) pAb-HRP (330, MBL) were employed as secondary antibodies. Finally, an enhanced chemiluminescence (ECL) immunoblotting detection kit (180-506, Tanon, Shanghai, China) was used for signal detection. The experiments were repeated three times, and ImageJ software was utilized to quantify the relative protein levels.

### 4.12. RNA-Seq and Data Analysis

Two-week-old seedlings of the wild type (Dongjin) and *SDG703* overexpressing lines, with or without infestation by SBPH for 12 h, were collected for RNA-seq analysis. Three biological replicates of each sample were submitted to BGI Genomics (The Beijing Genomics Institute) for RNA extraction, library construction, and RNA sequencing. High-throughput sequencing was performed using the BGISEQ-500 platform (BGI, Shenzhen, China) with 150 bp paired-end reads. Data analysis was conducted using the Dr. Tom system from BGI http://report.bgi.com (accessed on 10 July 2020). Differentially expressed genes were identified based on an adjusted *p*-value < 0.05 and fold-change > 2. The results were further validated by RT-qPCR.

### 4.13. Chromatin Immunoprecipitation Assays

Chromatin immunoprecipitation (ChIP) assays were conducted according to a previously published protocol [65]. For the ChIP experiments, a commercial antibody against H3K9me2 (Ab1220, Abcam) was employed. Two-week-old rice seedlings, cultivated in a growth chamber under LD conditions, were used for the ChIP assays. The enrichment of immunoprecipitated DNA in the ChIP experiments was determined by RT-qPCR. The primers listed in Table S1 were utilized to quantify the DNA levels from the ChIP products. Each cultivar was tested in three replicates.

### 4.14. Statistical Analysis

Statistical analyses were conducted using GraphPad Prism (Version 8.3.0) software. Differences between two groups were assessed using Student’s unpaired two-tailed *t*-tests. For multiple comparisons, when standard deviations were equal, two-way ANOVA (nonparametric) tests with Tukey’s multiple comparisons were employed.

### 4.15. Accession Numbers

Sequence data from this article can be accessed at the National Center for Biotechnological Information (NCBI) with the following accession numbers: *D53* (*Os11g0104300*), *OsWAK2-like* (*Os04g0370100*), *OsWAK3-like 1* (*Os11g0690332*), *OsWAK3-like 2* (*Os04g0367000*), *OsRLP7* (*Os12g0222900*), *OsRLP23* (*Os12g0221700*), *OsLecRK IX.1* (*Os08g0124100*), *OsXa21-like* (*Os11g0692500*), *OsJRL9* (*Os04g0330200*), *OsCRK44-like* (*Os04g0369033*), *OsRSR1* (*Os11g0229500*), *OsRGA5-like 1* (*Os11g0685600*), *OsRGA5-like 2* (*Os11g0588600*), *OsPIK-2-like* (*Os11g0598300*), *OsPIK-5-like* (*Os11g0689000*), *OsRPP13-like 1* (*Os01g0781600*), *OsWRKY53-like* (*Os11g0685700*), and *UBQ10* (*Os02g0161900*). The sequence data generated in this study have been deposited in the SRA database at NCBI with the corresponding accession numbers.

## 5. Conclusions

In conclusion, our findings unveil a novel role of SDG703 in regulating rice resistance against SBPH. Our results indicate that SDG703 is involved in mediating histone H3K9 methylation at the loci of PRRs and NLRs in rice, resulting in decreased resistance to SBPH. This study provides compelling evidence for the epigenetic regulation of plant–insect interactions, thereby enhancing the comprehension of the intricate regulatory mechanisms involving NLRs and PRRs in the resistance response against insect infestation.

## Data Availability

The data presented in this study are available upon request from the corresponding author.

## Supplementary Materials

The supporting information can be downloaded at: https://www.mdpi.com/article/10.3390/ijms241613003/s1.
