# Supplementary material for: SET Domain Group 703 Regulates Planthopper Resistance by Suppressing the Expression of Defense-Related Genes
Source: Int J Mol Sci. 2023 Aug 21;24(16):13003. doi: 10.3390/ijms241613003 (PMC10455402; doi:10.3390/ijms241613003)
Supplement: Supplementary file 1 [file ijms-24-13003-s001.zip › ijms-2523303-supplementary.pdf]

Supplementary Materials

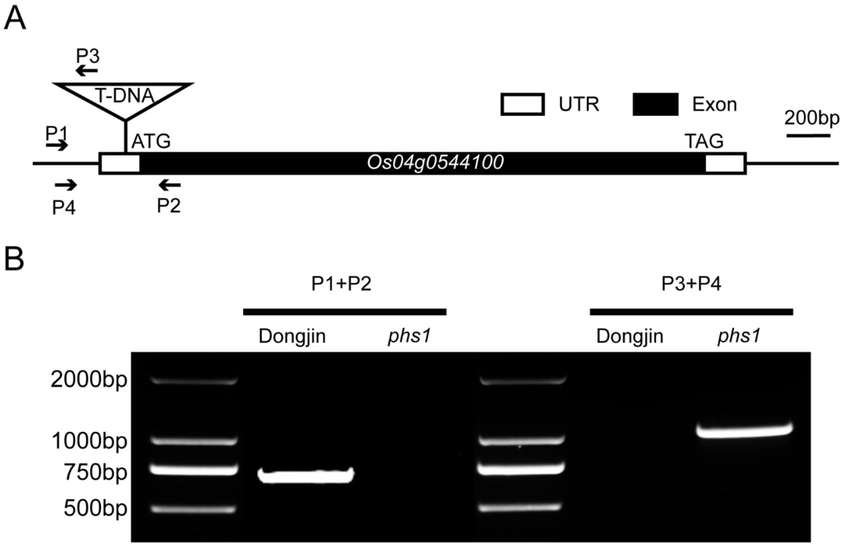

Figure S1. Analysis of mutant *phs1*. (**A**) Schematic representation of T-DNA insertion in the mutant *phs1* and the PCR primer position within the *SDG703* gene organization structure. Untranslated region (UTR), chromosome, and exon are represented by the white box, line, and black box, respectively. Forward and reverse PCR primers (labeled arrowed) served in genotyping PCR for Dongjin and mutant *phs1*, translation start codon ATG, and T-DNA insertion (triangle) are indicated in schematic representation. (**B**) T-DNA insertion verity of Dongjin and mutant *phs1* by PCR. Primers 1 and 2 amplified the 684 bp fragment only in Dongjin which have no T-DNA insertion. Primers 3 and 4 amplified the 1165 bp fragment only in mutant *phs1* which have T-DNA insertion.

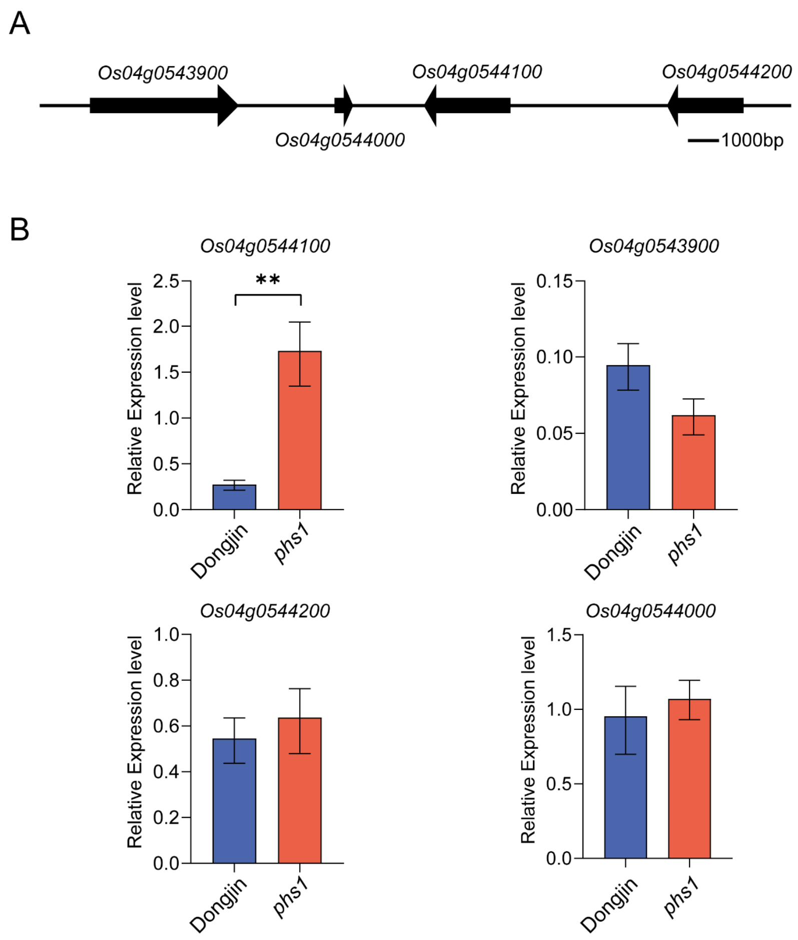

Figure S2. RT-qPCR analysis of mutant *phs1*. (**A**) Schematic representation of the arrangement of *SDG703* and its upstream and downstream genes on chromosomes. (**B**) RT-qPCR analysis of *SDG703* and its upstream and downstream genes in wild-type Dongjin and mutant *phs1* grown under normal condition, with *18s-rRNA* as the internal reference gene, n=3. Significant differences were determined using Student's *t*-test. ***P*<0.01.

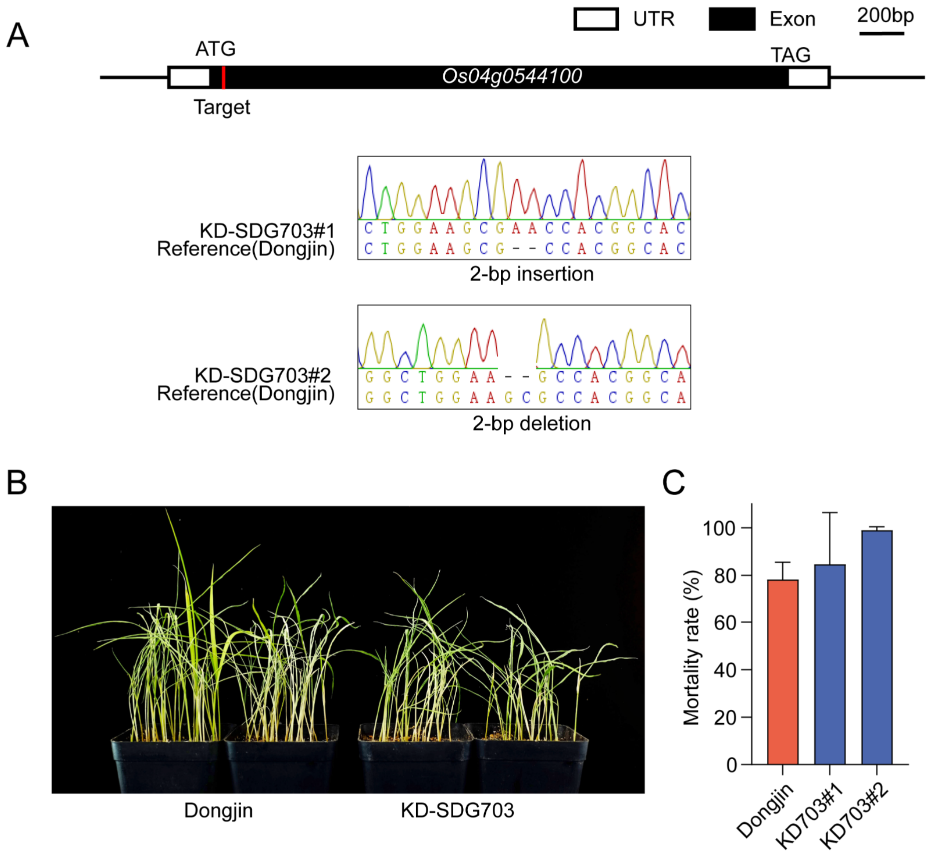

Figure S3. SBPH resistance phenotype of SDG703 knock-out transgenics. (**A**) SDG703 knock-out mutant *KD-SDG703* plants were screened by sequencing before being used in this study. The *KD-SDG703#1* harbors a 2-bp insertion in the exon and the *KD-SDG703#2* has a 2-bp deletion in the exon. (**B**) Phenotypes of wild-type and two independent SDG703 knock-out transgenic lines (*KD-SDG703#1*, *KD-SDG703#2*) subjected to SBPH infestation. Bar: 5cm. (**C**) The seeding mortality rate of wild-type and two independent *KD-SDG703* transgenics (n=3). Significant differences were determined using Student's *t*-test.

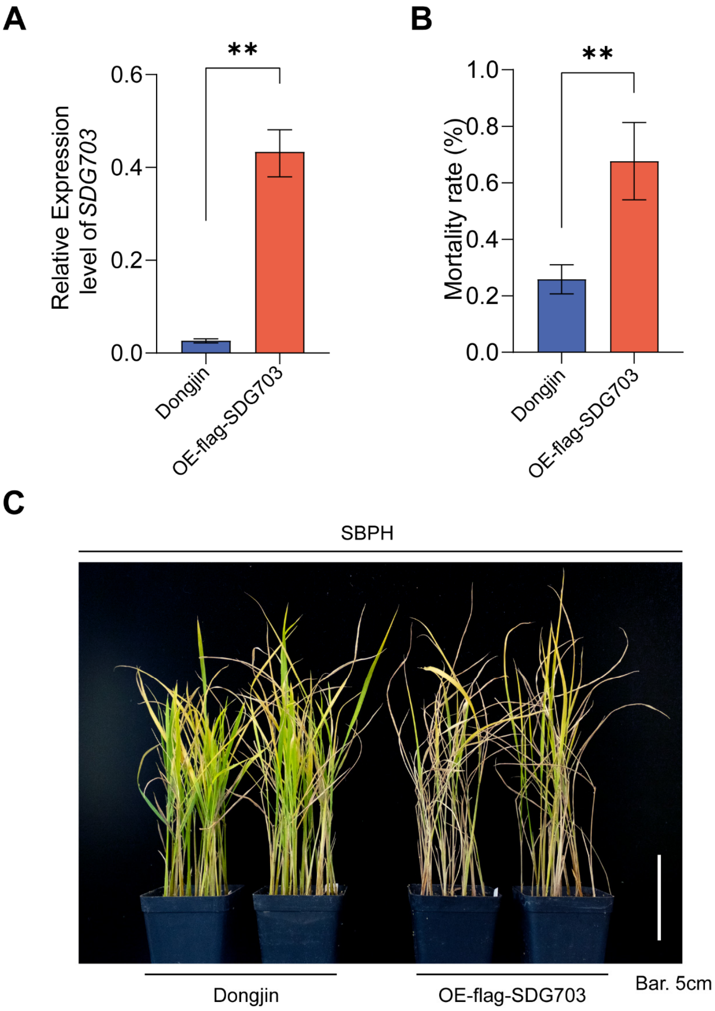

Figure S4. SBPH resistance phenotype of *OE-flag-SDG703*. (**A**) RT-qPCR analysis of SDG703 in wild-type Dongjin and *OE-flag-SDG703* grown under normal condition, with *18s-rRNA* as the internal reference gene. n=3. (**B**) Seeding mortality rate of wild-type and *OE-flag-SDG703*. n=3. Significant differences were determined using Student's *t*-test. ***P*<0.01. (**C**) Phenotypes of wild-type and transgenic line plants *OE-flag-SDG703* subjected to SBPH infestation. Bar: 5cm.

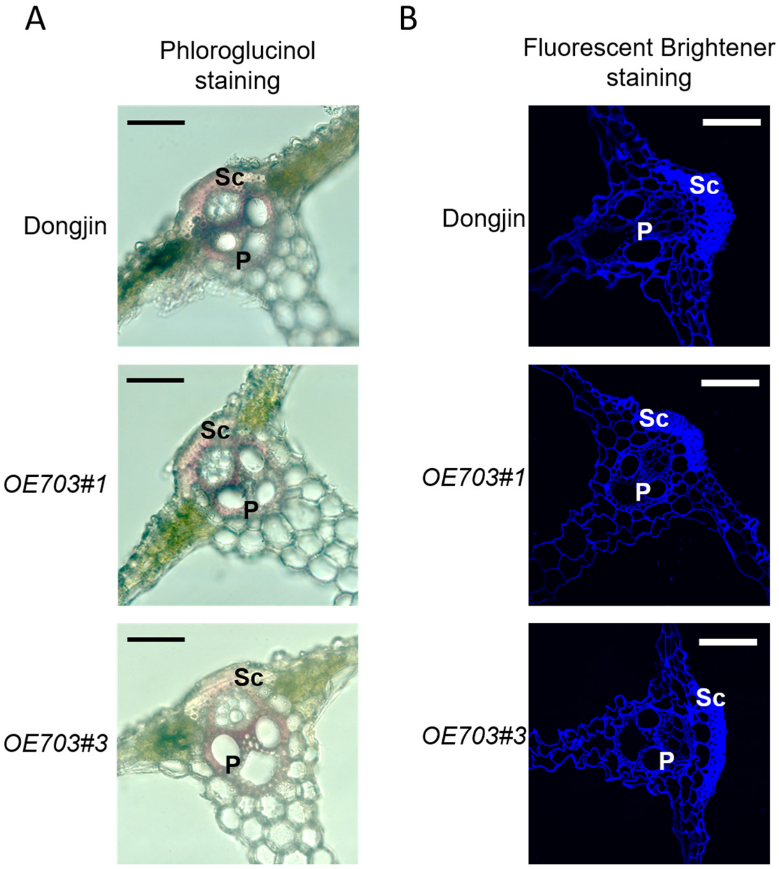

Figure S5. SDG703 has no relationship with lignin and cellulose accumulation in rice sheaths. (A) Representative images of lignin accumulation showed by phloroglucinol staining in fresh leaf sheaths of wild-type Dongjin and OE-SDG703. Sc: sclerenchyma; P: phloem. Scale bar: 50 µm. (B) Representative images of cellulose accumulation showed by fluorescent brightener staining in the semithin section of leaf sheaths in wild-type and OE-SDG703. Sc: sclerenchyma; P: phloem. Scale bar: 50 µm.

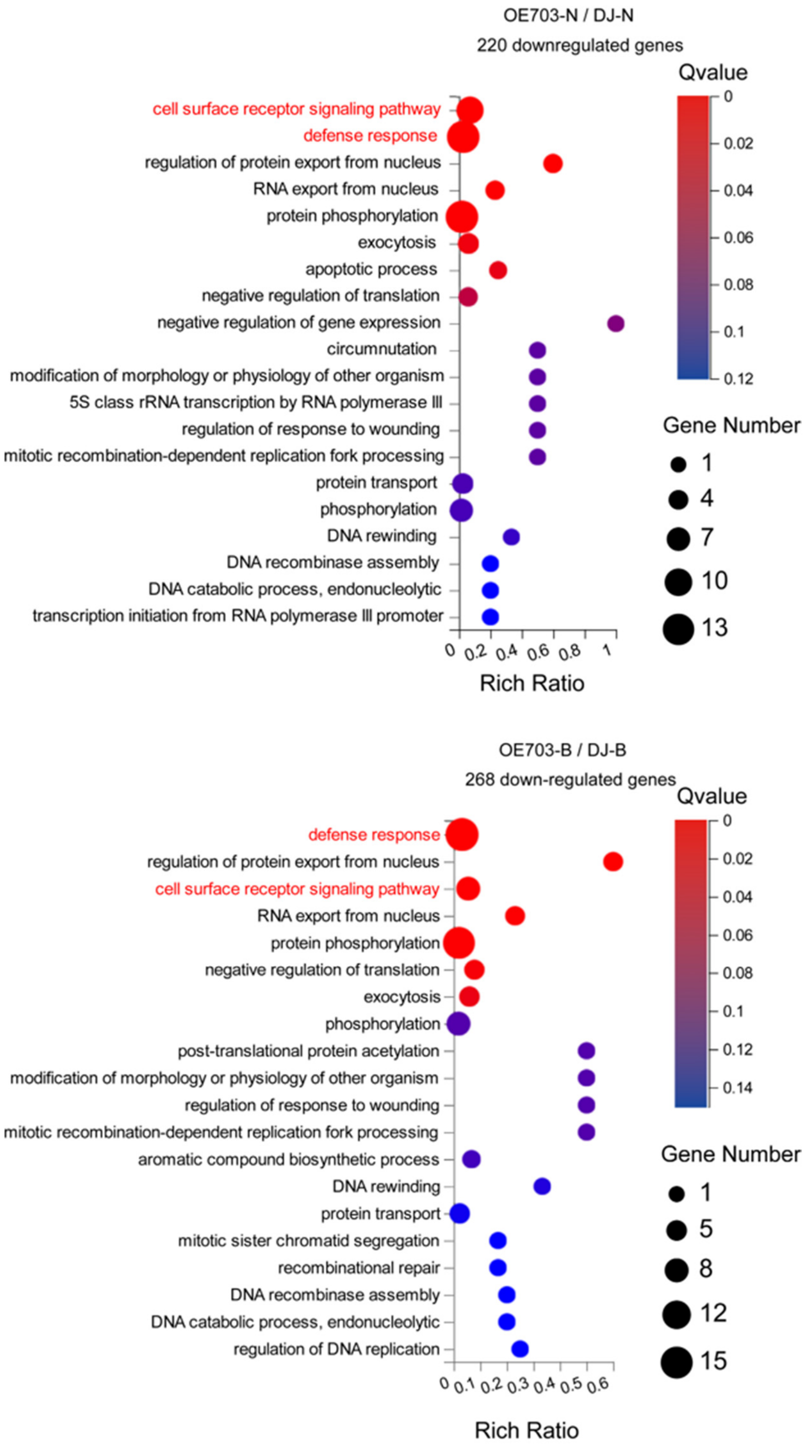

Figure S6. Gene ontology (GO) analysis of downregulated genes in *OE-SDG703* lines compared with wild-type Dongjin in normal condition or SBPH infestation. The size of each point represents the number of genes, and Qvalue are indicated with various colors.

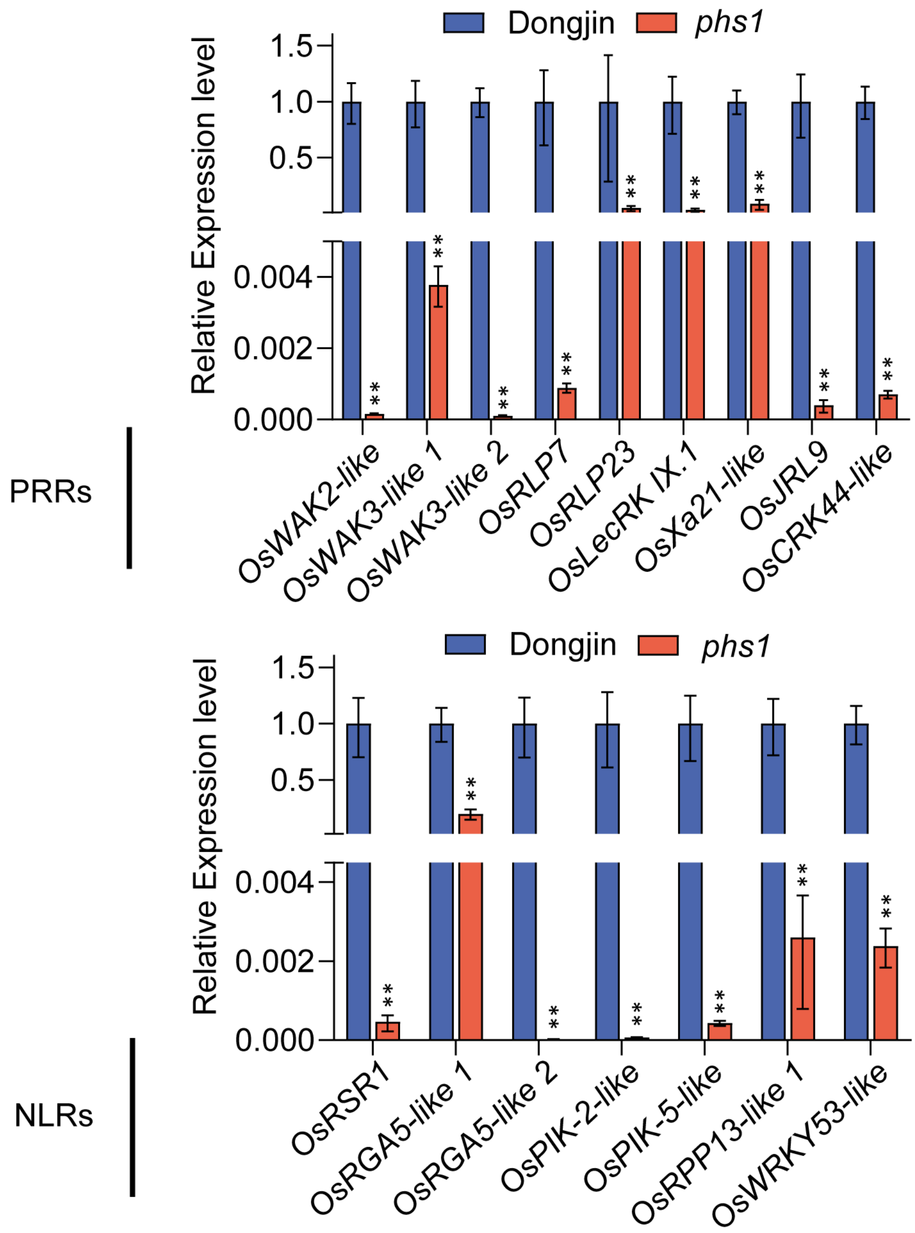

Figure S7. Expression analysis of PRR and NLR genes in wild-type and mutant *phs1* with normal condition by RT-qPCR. Values are the means ± SD of three individual biological replicates normalized to the internal control *18s-rRNA*. Significant differences were determined using Student's *t*-test. ***P*<0.01.

Table S1. List of nucleotide sequences of primers used in this study.

Primer usage	Primer name	Forward primer sequence（5'~3'）	Reverse primer sequence（5'~3'）
Verification of *phs1*	P1	CAGTCCGACTCCGATCCA	
	P2		CCCTGCCATTCCTCAAAC
	P3	CCGACTCCGATCCACCAT	
	P4		TCGGCTTCAAATGGCGTA
	flanking-sequence-14509	TATTGACCCACACTTTGCCGTA	CACCAGAAGCCTCTAACCCT
Vector Construction	Crispr-SDG703-33	GGCAAAGCCCGGCTGGAAGCGCCA	AAACTGGCGCTTCCAGCCGGGCTT
	pAN580-SDG703	CGGAGCTAGCTCTAGAATGGGGATCCCGGAGG	TGCTCACCATGGATCCATACAACCAACCATCAC
	pCambia1300-SDG703-Flag	ACGATGATAAGGGCGGTACCATGGGGATCCCGGAGGTG	TGTAGTCCATGTCGACATACAACCAACCATCAC
	pCambia1305-SDG703-GFP	CGGAGCTAGCTCTAGAATGGGGATCCCGGAGG	TGCTCACCATGGATCCATACAACCAACCATCAC
	pCambia1390-SDG703	TTACTTCTGCACTAGGTACCATGGGGATCCCGGAGGTGGT	GAATTCCCGGGGATCCCTAATACAACCAACCATCAC
	SDG703pro	GAATTCCCGGGGATCCAAGTCTACTCGCTAGGAATTTCG	GGCCAGTGCCAAGCTTCCAACGCCCCCCGCACT
RT-qPCR	RT_SDG703	CCAACTGATCCAGAAGCTGA	TTCTCCTCATCCCACATGAA
	RT_OsGDH2	CCAACTGATCCAGAAGCTGA	TTCTCCTCATCCCACATGAA
	RT_OsCYCP4	TACAACAACGCCTACTTCGC	CAGTAGTGGCCGAACGTCT
	RT_Os04g45980	GGCAACTGAAACTCTGAAACC	AGTAGGTAACAAAGCTCAACCA
	18S-rRNA	ATGATAACTCGACGGATCGC	CTTGGATGTGGTAGCCGTTT
	RT_OsRSR1	CTTTAAGATGCATGACCTCGTG	ACGAACTTCCTTATCCATCCTC
	RT_OsJRL9	AAACGTGTGAAAGGTACCCTG	CATGTGCGTCAACACCTCC

Primer usage	Primer name	Forward primer sequence（5'~3'）	Reverse primer sequence（5'~3'）
	RT_OsWAK3-L1	CGAGAAAGGCTGGTACAATTTC	TCAAGTACAGTTGGGACACTTT
	RT_OsWAK3-L2	CAACATACTTTGGATATCTGTGC	TTTTCTGGCAAAACAACCTTC
	RT_Xa21-L	CCTTCTTTTGCCAGTATTCGAG	CAAGTGAGATAGTGTCCGCATA
	RT_OsRLP7	GCCATGCTACTCATCCTCGT	CTACTGCTTCCGTGGAGCTT
	RT_OsRLP23	TACCAGTTTACTGCTGCACTAA	GTCCAAAGACTCAAGGTTGTTC
	RT_OsLecRK IX.1	CAGCTGCATTCATGGTATTTCA	CATCTCGATCTCTACGATGGTC
	RT_OsCRK44-L	GCTTGGATGCAAAGATATCAACGA	CTTTCGGATAACCCGGAGGG
	RT_OsWAK2-L	GAAGTTTCGATTGTGTGTGTCA	GTCGTGTTTTCGGTGATAAGAG
	RT_OsWRKY53-L	GATGGTCGACGTCGTATACAAG	ATACACCGGTAGTAGCATCTTG
	RT_OsRPP13-L	ACTCTCTCTTTCCTCCTGTAGT	CCAACTGTAAAGAAGGACAGGA
	RT_PIK-5-L	AAATTCCAACCACTAGCCAAAC	ATTCCAATCTGAATTAGGCCGA
	RT_Pik-2-L	GAGGAGATGGAGAGCATGAAC	GAGTCTACCTTTGGTACGGTG
	RT_OsRGA5-L1	ATGTTGATGTTCTCGGAGGATT	AACTCCAGACAGATGTTCGATT
	RT_OsRGA5-L2	ATGGTTAATTGACAAGTGCCAC	GCGACATCATCAATCTTGTACC
	ChIP_WAK3-L-P1	GCACATTCAAGCAACACCA	GCATCACTCCTTGTGCCATG
	ChIP_WAK3-L-P2	AACCATAGCTTTACCCCTCCC	TGCCTCCACCGAGATGTCC
	ChIP_WAK3-L-P3	GTACCAACTAATGCGTGCC	CTTTAAATAAGACGGACGGTT
	ChIP_WAK3-L-P4	TTACTCTTATAATTTCGTCCC	TAGATTAGTATTGCCATCGAC
ChIP-qPCR	ChIP_WAK3-L-P5	ATAAGCAAAATCACAGATCGC	TCCCCTATAGACCTTACCGAA
	ChIP_Xa21-L-P1	CAACGCTTATATACCAGGAAC	CCATAGAAGCATTACGCAGA
	ChIP_Xa21-L-P2	TGCCATTTTCAACATGTCTAGCC	CCCGTCAGATTGTTATTCCAGA
	ChIP_Xa21-L-P3	ACAGGACTAATCCCGTCAGG	AAATTGTCTCAAGATTCTGGC
	ChIP_Xa21-L-P4	TTCACAGATTCAATCCCGAAC	TCCAATACTTCTAAGCTGGTC

Primer usage	Primer name	Forward primer sequence（5’~3’）	Reverse primer sequence（5’~3’）
	ChIP_Xa21-L-P5	TGCTTCAGTATATGCCCAA	TGTGCAAGTACGTCTCCAA
	ChIP_RLP7-P1	CAGGCAATATTGTATCCATG	AAAGACAAGTTGATAGGCAA
	ChIP_RLP7-P2	TCGGTGACTACTCTGCCGCCTT	CCGTCCCAGCTGCAACAGT
	ChIP_RLP7-P3	GCCGCCTCACCAGACTCTCC	GCTGTACTCGTCGTCCAACTCCT
	ChIP_RLP23-P1	TAGACGTACCTGTGCTTATCCG	TTTGCAACTCTATGGCACAC
	ChIP_RLP23-P2	CGCATTGTTCAGCCTGACC	AGATGTCAAGGTACTCCAGC
	ChIP_RLP23-P3	TATTGCTCACTTTCTGGTCCG	ACAACAAGTGATTTCAAGGCAG
	ChIP_RLP23-P4	TGCCTTCTTCCATTGGTAAGCTG	ATCCTACTAGCTCCAACCCAGA
	ChIP_RLP23-P5	ATATAACCAGATCAGAGGTGCAA	TAGCCTGAGGTCTTCCATACCC
	ChIP_RLP23-P6	CTGCACTAACATACAAAGGCAAT	TGAGACATCAATCAGCACGAG
	ChIP_JRL9-P1	GTAAACTACAATCCGTTGCT	TGACAGCCATTATTTAACCC
	ChIP_JRL9-P2	ATGTTGTTTGAATACGACGA	GTACCTTTCACACGTTTGC
	ChIP_JRL9-P3	GTTTTAAATGAACGGACACCT	TTAGCGCATCATTACTGCAA
	ChIP_JRL9-P4	TAACCTGATGCATGGACGCACAC	CATGTGCGTCAACACCTCCC
	ChIP_JRL9-P5	CGGCTACCATGACAACCAC	TGAACTGCAACCACCGGAT
	ChIP_RSR1-P1	AAACCTTTTATAACGGAGCAT	ATGTCCATATGTGTTCCGTA
	ChIP_RSR1-P2	CGGAGATTTGAGGCTAGTGGA	GGCGAGAGACGACCAGAGTA
	ChIP_RSR1-P3	TCCCTCCTCTTTGGGCTGA	ACATATTATATGCCACGGTCGAA
	ChIP_RSR1-P4	ATATTTCTAAAATGACCAGCAG	TCCAAAGATTGTATCGCTTG
	ChIP_RSR1-P5	AATCCAGAACTGATATCGC	CAATTTGACCGTATTTACACC
	ChIP_RSR1-P6	CGTATGCCTGATGTAAACCAC	ACCTAAGAGACTCAATGCCTT
	OsUBQ10-A	TCAGGATACGTGTGCTGTTACTACC	ACAGCAAACATATTCCAACAGAACA
	OsUBQ10-B	GATGGCAGGACCCTTGCTGACTA	CTGGATCTTGGCCTTGACGTTGT

Table S2. Fifty three candidate genes in Venn diagram

Gene Symbol	Chr.	DJ_N Average FPKM	DJ_B Average FPKM	OE703_N Average FPKM	OE703_B Average FPKM	GeneBank Desc
LOC4343276	chr7	1.31	6.16	0.09	0.16	ATP-dependent DNA helicase SRS2-like protein At4g25120
LOC4351801	chr12	1.46	4.48	0.00	0.00	chitinase-like protein 1
LOC4335481	chr4	7.91	19.57	0.10	0.42	cysteine-rich receptor-like protein kinase 6
LOC4350071	chr11	1.91	8.04	0.00	0.00	dirigent protein 1
LOC4351960	chr12	2.35	11.94	0.14	1.00	disease resistance protein Pik-2-like
LOC4350850	chr11	5.37	10.74	0.00	0.00	disease resistance protein Pik-2-like
LOC107278320	chr11	0.55	1.55	0.00	0.00	disease resistance protein PIK5-NP-like
LOC112936146	chr11	9.76	26.29	1.01	1.61	disease resistance protein RGA5-like
LOC9267585	chr11	11.13	26.25	0.00	0.00	disease resistance protein RGA5-like
LOC4350133	chr11	5.18	23.70	0.01	0.01	disease resistance protein RPM1
LOC4338575	chr5	0.30	1.05	0.00	0.01	exocyst complex component EXO70B2
LOC4343472	chr7	1.48	4.13	0.09	0.14	F-box protein At5g07610
LOC4352868	chr12	246.54	2406.69	54.60	381.62	glycine-rich RNA-binding protein 2
LOC4335495	chr4	18.62	46.99	0.00	0.00	jacalin-related lectin 9
LOC4344556	chr8	0.93	3.84	0.05	0.01	L-type lectin-domain containing receptor kinase IX.1
LOC4350701	chr11	2.69	7.02	0.30	1.01	maf-like protein DDB_G0281937
LOC9271010	chr12	1.44	4.99	0.02	0.02	phosphoglycolate phosphatase 1B, chloroplastic
LOC4351165	chr11	1.18	11.52	0.27	0.44	probable LRR receptor-like serine/threonine-protein kinase At3g47570
LOC107279538	chr11	0.08	0.39	0.00	0.00	probable LRR receptor-like serine/threonine-protein kinase At3g47570
LOC9272184	chr12	4.59	11.51	0.00	0.00	probable staphylococcal-like nuclease CAN4
LOC107276072	chr11	12.32	36.34	0.19	0.00	probable WRKY transcription factor 53

Gene Symbol	Chr.	DJ_N Average FPKM	DJ_B Average FPKM	OE703_N Average FPKM	OE703_B Average FPKM	GeneBank Desc
LOC107281147	chr5	2.77	5.70	0.30	1.06	protein FAR1-RELATED SEQUENCE 5
LOC9268622	chr1	0.83	2.32	0.01	0.00	putative disease resistance RPP13-like protein 1
LOC4345291	chr8	0.87	2.70	0.01	0.04	putative F-box/LRR-repeat protein At5g41630
LOC4335588	chr4	2.43	5.64	0.02	0.75	putative receptor-like protein kinase At4g00960
LOC107279563	chr12	0.18	1.09	0.02	0.14	receptor like protein 23
LOC4351803	chr12	0.51	3.92	0.00	0.00	receptor-like protein 7
LOC4351209	chr11	2.78	8.62	0.43	1.93	selenoprotein H
LOC4328654	chr2	12.89	28.15	0.64	2.42	SPX domain-containing protein 2-like
LOC4350688	chr11	23.56	48.83	1.89	3.35	ubiquitin carboxyl-terminal hydrolase 18
LOC107276434	chr12	0.61	4.96	0.00	0.00	uncharacterized LOC107276434
LOC107277588	chr2	0.09	0.44	0.00	0.00	uncharacterized LOC107277588
LOC4329988	chr2	3.03	9.52	0.00	0.00	uncharacterized LOC4329988
LOC4335494	chr4	0.41	1.95	0.00	0.11	uncharacterized LOC4335494
LOC4338795	chr5	0.22	1.05	0.02	0.04	uncharacterized LOC4338795
LOC4340728	chr6	3.09	11.23	0.00	0.00	uncharacterized LOC4340728
LOC4342178	chr7	1.47	4.92	0.00	0.00	uncharacterized LOC4342178
LOC4343370	chr7	0.42	1.50	0.01	0.01	uncharacterized LOC4343370
LOC4350122	chr11	0.69	2.21	0.00	0.00	uncharacterized LOC4350122
LOC4350599	chr11	9.84	20.63	1.63	3.34	uncharacterized LOC4350599
LOC4351136	chr11	3.70	13.33	0.39	0.43	uncharacterized LOC4351136
LOC4351893	chr12	18.76	47.06	0.00	0.02	uncharacterized LOC4351893
LOC4352075	chr12	4.69	14.20	0.01	0.00	uncharacterized LOC4352075

Gene Symbol	Chr.	DJ_N Average FPKM	DJ_B Average FPKM	OE703_N Average FPKM	OE703_B Average FPKM	GeneBank Desc
LOC4352102	chr12	10.28	23.99	0.03	0.05	uncharacterized LOC4352102
LOC9266148	chr12	1.94	6.79	0.00	0.00	uncharacterized LOC9266148
LOC9266760	chr11	1.01	3.80	0.00	0.00	uncharacterized LOC9266760
LOC9267533	chr11	2.44	5.89	0.02	0.03	uncharacterized LOC9267533
LOC9270995	chr5	0.27	1.95	0.07	0.20	uncharacterized LOC9270995
LOC9272159	chr10	3.64	13.49	0.01	0.01	uncharacterized LOC9272159
LOC4350669	chr11	1.01	3.45	0.00	0.00	UPF0481 protein At3g47200
LOC9272252	chr4	2.24	5.54	0.00	0.00	wall-associated receptor kinase 2
LOC107278106	chr4	7.00	14.69	0.05	0.14	wall-associated receptor kinase 3
LOC107276842	chr11	1.31	14.51	0.00	0.01	wall-associated receptor kinase 3

Table S3. List of differentially expressed genes (DEGs) in *SDG703-OE* vs. Dongjin which was selected for validation by RT-qPCR.

	Gene Symbol	RAP_Locus	GeneBank Desc
PRRs	LOC107276842	*Os11g0690332*	wall-associated receptor kinase 3
	LOC4351165	*Os11g0692500*	probable LRR receptor-like serine/threonine-protein kinase At3g47570
	LOC4351803	*Os12g0222900*	receptor-like protein 7
	LOC107279563	*Os12g0221700*	receptor like protein 23
	LOC4344556	*Os08g0124100*	L-type lectin-domain containing receptor kinase IX.1
	LOC4335495	*Os04g0330200*	jacalin-related lectin 9
	LOC4335481	*Os04g0322100*	cysteine-rich receptor-like protein kinase 6
	LOC9272252	*Os04g0370100*	wall-associated receptor kinase 2
	LOC4335588	*Os04g0369033*	putative receptor-like protein kinase At4g00960
	LOC107278106	*Os04g0367000*	wall-associated receptor kinase 3
NLRs	LOC4350133	*Os11g0229500*	disease resistance protein RPM1
	LOC107276072	*Os11g0685700*	probable WRKY transcription factor 53
	LOC107278320	*Os11g0689000*	disease resistance protein PIK5-NP-like
	LOC9268622	*Os01g0781600*	putative disease resistance RPP13-like protein 1
	LOC112936146	*Os11g0685600*	disease resistance protein RGA5-like
	LOC9267585	*Os11g0588600*	disease resistance protein RGA5-like
	LOC4350850	*Os11g0598300*	disease resistance protein Pik-2-like
